# Comments on: Usefulness of indocyanine green fluorescence imaging to evaluate intestinal blood flow during laparoscopic surgery for strangulated small bowel obstruction: a report of two cases

**DOI:** 10.1016/j.ijscr.2025.112012

**Published:** 2025-10-10

**Authors:** Muhammad Talha Ali, Aakash Kabir, Hassnain raza

**Affiliations:** a Liaquat College of Medicine & Dentistry, Street-19, Block 15, KDA Scheme No. 36, Gulistan-e-Jauhar, Karachi, Pakistan; b Liaquat College of Medicine & Dentistry, Pakistan

Letter to the Editor

The article titled “Usefulness of Indocyanine Green Fluorescence Imaging to Evaluate Intestinal Blood Flow during Laparoscopic Surgery for Strangulated Small Bowel Obstruction: A Report of Two Cases” by Noguchi R, Suda K, Watanabe K, et al. [1] is most impressive in its application of indocyanine green fluorescence imaging in ICG-FI during emergency laparoscopic surgeries to improve outcomes by limiting unnecessary bowel resections. we appreciate the conclusion the authors have arrived at, but to strengthen the clinical applicability of their findings, we would like to offer several additional considerations.

To begin, in both cases, intraoperative decision-making hinged upon qualitative visual assessments of fluorescence without evaluating onset times and signal intensities, which previous studies have associated, albeit indirectly, with bowel viability [2,3]. This addition could minimize inter-surgeon variability.

Second, the document does not provide histopathological validation of ICG-FI findings, which is a significant omission. Although ICG-FI is capable of visualizing macrovascular perfusion but is not effective for microvascular circulation, an area with preserved fluorescence could still suffer from compromised microvascular circulation, which is a potential source of postoperative complications [4].

Third, although limited by the case report format, the absence of long-term postoperative outcomes is notable. Following up within a 30–90 day postoperative timeframe via imaging or endoscopy could have yielded valuable information on the preservation of bowel anastomosis.

In the fourth instance, the issue of the cost-effectiveness of the procedure was not explored. Although the procedure may be associated with a reduction in morbidity due to the avoidance of bowel resection, the near-infrared imaging systems (often exceeding $50,000) may pose a limitation in terms of the cost-to-benefit balance in the global south.

In the final instance, the role of ICG-FI could have benefited from a cursory assessment of other intraoperative perfusion assessment devices, such as laser Doppler flowmetry, which can assess microcirculatory flow or pulse oximetry.

In conclusion, this case report offers valuable insight into the emerging role of ICG-FI in emergency gastrointestinal surgery. If future investigations perform histological validation, long-term outcome assessments, cost-benefit analyses, comparisons to alternative techniques, and the application of quantitative fluorescence, ICG-FI will have much stronger evidence for this clinical application.

## Author disclosure

This letter is an independent commentary on article published by Ryota Noguchi et al. and does not involve any new data collection or analysis.

## Consent

Not applicable this Letter to the Editor does not include any patient information, images, or case details requiring consent.

## Ethical approval

This article is a Letter to the Editor and does not involve human participants, animals, or patient data. Therefore, ethical approval was not required. Institution: Liaquat College of Medicine & Dentistry, Karachi, Pakistan.

## Guarantor

Muhammad Talha Ali accepts full responsibility for the work, had access to all materials used in its preparation, and controlled the decision to publish.

## Registration of research studies

Not applicable. this submission is a commentary/Letter to the Editor and does not involve new human studies.

## Funding

No funding was received for the preparation of this letter.

## Declaration of competing interest

The authors declare no conflicts of interest related to this letter.
